# Diurnal Variation of Hormonal and Lipid Biomarkers in a Molecular Epidemiology-Like Setting

**DOI:** 10.1371/journal.pone.0135652

**Published:** 2015-08-18

**Authors:** Linda W. M. van Kerkhof, Kirsten C. G. Van Dycke, Eugene H. J. M. Jansen, Piet K. Beekhof, Conny T. M. van Oostrom, Tatjana Ruskovska, Nevenka Velickova, Nikola Kamcev, Jeroen L. A. Pennings, Harry van Steeg, Wendy Rodenburg

**Affiliations:** 1 Centre for Health Protection, National Institute for Public Health and the Environment (RIVM), Bilthoven, The Netherlands; 2 Department of Genetics, Center for Biomedical Genetics, Erasmus University Medical Center, Rotterdam, The Netherlands; 3 Faculty of Medical Sciences, Goce Delcev University, Stip, Republic of Macedonia; 4 Department of Human Genetics, Leiden University Medical Center, Leiden, The Netherlands; University of Alabama at Birmingham, UNITED STATES

## Abstract

**Introduction:**

Many molecular epidemiology studies focusing on high prevalent diseases, such as metabolic disorders and cancer, investigate metabolic and hormonal markers. In general, sampling for these markers can occur at any time-point during the day or after an overnight fast. However, environmental factors, such as light exposure and food intake might affect the levels of these markers, since they provide input for the internal time-keeping system. When diurnal variation is larger than the inter-individual variation, time of day should be taken into account. Importantly, heterogeneity in diurnal variation and disturbance of circadian rhythms among a study population might increasingly occur as a result of our increasing 24/7 economy and related variation in exposure to environmental factors (such as light and food).

**Aim:**

The aim of the present study was to determine whether a set of often used biomarkers shows diurnal variation in a setting resembling large molecular epidemiology studies, i.e., non-fasted and limited control possibilities for other environmental influences.

**Results:**

We show that markers for which diurnal variation is not an issue are adrenocorticotropic hormone, follicle stimulating hormone, estradiol and high-density lipoprotein. For all other tested markers diurnal variation was observed in at least one gender (cholesterol, cortisol, dehydroepiandrosterone sulfate, free fatty acids, low-density lipoprotein, luteinizing hormone, prolactin, progesterone, testosterone, triglycerides, total triiodothyronine and thyroid-stimulating hormone) or could not reliably be detected (human growth hormone).

**Discussion:**

Thus, studies investigating these markers should take diurnal variation into account, for which we provide some options. Furthermore, our study indicates the need for investigating diurnal variation (in literature or experimentally) before setting up studies measuring markers in routine and controlled settings, especially since time-of-day likely matters for many more markers than the ones investigated in the present study.

## Introduction

Many molecular epidemiology studies focusing on high prevalent diseases, such as metabolic disorders and cancer [1, 2], make use of metabolic and hormonal markers (for examples see references [3, 4]. Metabolic markers might serve as important (early) indicators for metabolic disorders, including cardiovascular diseases and type 2 diabetes, and hormonal disbalance is often studied in large epidemiological settings since these are associated with high incidence cancers, such as breast cancer [5–7]. In general, for these markers sampling can occur at any time-point during the day or after an overnight fast. However, environmental factors, such as light exposure and food intake might affect the levels of these markers, since they provide input for the internal time-keeping system [8–11]. Several markers are well known for their diurnal variation, for example cortisol and melatonin [12]. Importantly, heterogeneity in diurnal variation and disturbance of circadian rhythms among a study population might increasingly occur as a result of our increasing 24/7 economy and related variation in exposure to environmental factors (such as light and food). For example, previous studies have indicated that there are substantial changes in the blood transcriptome after experiencing insufficient or mistimed sleep [13, 14]. This might render accurate determination of markers in molecular epidemiology studies challenging. When the diurnal variation is larger than the inter-individual variation, time of day should be taken into account in molecular epidemiology studies. In this study, we determine whether a set of biomarkers relevant for metabolic disorders and hormone-associated cancers, consisting of endocrine and sex hormones and lipids, shows diurnal variation.

The diurnal variation of various blood markers has previously been studied in different settings, ranging from a relatively uncontrolled routine setting to very tightly controlled laboratory settings. Most information on diurnal variation is derived from these controlled laboratory studies in which factors influencing diurnal variation (such as food intake and/or sleep/wake cycle) are controlled (for example see references [15–21]). Due to the large-scale set up of some molecular epidemiology studies, the possibility for standardization of sample collection to time of day and food consumption is limited. Therefore, most molecular epidemiology studies collect blood samples to study biomarkers for adverse health outcomes without standardization for time-point, food intake or sleep.

The aim of the present study was to investigate diurnal variation of a set of markers (relevant for metabolic disorders and hormone-associated cancers) in a routine setting in males and females, resembling molecular epidemiology studies: namely, non-fasted and limited control for other environmental influences. In addition, classical markers to study circadian rhythms in chronobiology research are measured: cortisol and components of the molecular biological clock i.e. ‘clock genes’. We determined cortisol levels and clock gene expression levels in blood throughout the day to study diurnal variation of these classical markers in a routine setting.

## Methods

### Study design and ethics statement

Approval for all procedures was obtained from the ethical committee of the Goce Delchev University in Stip, Republic of Macedonia. All participants signed an informed written consent. This was provided in Macedonian and the translation would be as follows: "I confirm that I am informed about the goals of the Circadian study and voluntary participate in it. I also confirm that all information that I provided with regards to my general health condition and lifestyle is correct”. Samples of 17 healthy volunteers, 10 women and 7 men were analyzed in this study. Inclusion criteria were:-age over 21 years old,—apparently healthy without any acute or chronic disease (general medical examination without laboratory analyses),—not taking any drugs, multivitamins and/or supplements, -non-smoking,—not more than 1 (for women) or 2 (for men) alcoholic beverages per day,—no shiftwork and have a normal circadian rhythm (sleeping at nighttime).

The mean age of the males was 24.9 ± 7.2 years and 21.7 ± 0.5 years for the female volunteers. The mean weight was 84.1 ±13.9 kg for the males and 58.5 ±13.6 kg for the females. For more participants characteristics and individual data, see S1 Table. The samples were collected around the clock at four-hour intervals. The first sample was collected at 08:00 AM after overnight fasting. Participants were asked when they slept between the first and last sampling times (8:00–4:00 AM). Total sleep duration between these time-points was 3.4 ± 0.9 hr for males and 3.2 ±1.9 for females. For individual sleeping duration and timing of sleep episodes, see S1 Table. Volunteers were asked to consume their meals one hour after sample collection and otherwise continue their normal daily routines. Meal timing and composition was not further restricted or standardized and meals were consumed at home. EDTA plasma and buffy coat samples (2.7 mL) and serum samples (7.5 mL) were collected in a routine clinical chemistry laboratory by venipuncture of the antecubital vein, using the commercial blood sampling method Sarstedt (Sarstedt AG & Co., Nümbrecht, Germany). Individuals were allowed to leave the laboratory after sample collection and thus were required to wake up and come to the laboratory for sample collection at night.

### Plasma and serum analyses

Two methods were used to determine hormonal and lipid biomarkers, an overview can be found in S2 Table. Free fatty acids (FFA), high-density lipoprotein (HDL), low-density lipoprotein (LDL), triglycerides (TG) and cholesterol (CHOL) were determined with an auto-analyzer (Unicel DxC 800, Beckman-Coulter, Woerden, the Netherlands), using kits from Beckman-Coulter. Cortisol (CORT), dehydroepiandrosterone sulfate (DHEAS), estradiol (E2), human growth hormone (hGH), prolactin (PRL), progesterone (PRG), testosterone (TEST), total triiodothyronine (TotT3), thyroid-stimulating hormone (TSH) were determined with an immune-analyzer (Access-2, Beckman-Coulter) using dedicated kits from Beckman-Coulter. Levels of adrenocorticotropic hormone (ACTH), follicle stimulating hormone (FSH), and luteinizing hormone (LH) were determined using commercially available Milliplex Kits (Millipore Corporation, Billerica, MA, USA). All measurements were performed on the same day. The intra-assay variation was determined with three quality control samples (8 tests) for markers measured on the auto-analyzer and with two quality control samples (5 tests) for markers measured on the immune-analyzer. The CVs of markers measured with the Luminex technique were obtained from the manufacturer. See S2 Table for all CVs.

### Blood RNA analysis

Expression levels of clock genes *BMAL1* and *PER1* were determined in buffy coats of a subset of volunteers (n = 6, female), using quantitative reverse transcription polymerase chain reaction (RT-PCR). All oligonucleotide primers were obtained from Life Technologies (Bleiswijk, The Netherlands). Total RNA was extracted from buffy coats stabilized with Qiazol, using the miRNeasy Mini Kit (both Qiagen Benelux, Venlo, The Netherlands). cDNA was made using the high capacity cDNA reverse transcription kit (Applied Biosystems by Life Technologies, Bleiswijk, The Netherlands). qPCR was performed on the 7500 Fast Realt-Time PCR System (Applied Biosystems by Life Technologies, Bleiswijk, The Netherlands) using Taqman Fast Universal PCR Master Mix and Taqman Gene Expression assays for *BMAL1* (Hs00154147_m1) and *PER1* (Hs01092603_m1) all according to protocol of manufacturer (Applied Biosystems by Life Technologies, Bleiswijk, The Netherlands).

### Statistics

To detect all types of diurnal variation, parameters were tested using two types of analyses: cosine fitting analysis and Repeated Measures ANOVA (RM-ANOVA). Depending on the shape of the day curves, these analyses can be overlapping and/or complementary. The RM-ANOVA detects effects of time, i.e. are the levels of the marker different among time-points. The cosine analysis enables detection of rhythms that follow a cosine curve, in manner that is occasionally more sensitive than the RM-ANOVA. For the cosine analysis, CircWave Batch v5.0 software (Roelof Hut, www.euclock.org) was used. A closer fit to cosine gives lower p-value, *p* < 0.05 is assumed a significant cosine curve fit. Repeated Measures ANOVA (RM-ANOVA) was performed using GraphPad Prism software version 6.04 for Windows (GraphPad Software, San Diego California USA, www.graphpad.com). The Greenhouse-Geisser epsilon indicated that sphericity could not be assumed in most analyses, therefore, Greenhouse- Geisser correction was applied. For the RM-ANOVA, *p* < 0.05 was considered statistically significant. If CircWave analysis, RM-ANOVA, or both indicated that signification variation was present, we identified the markers as having diurnal variation in our experiment. Gene expression of *PER1* and *BMAL1* was given as relative expression compared to the mean.

Considering the known difference in levels of several makers between genders, all statistical analyses were performed separately for males and females. The aim of this study was to identify markers with diurnal variation, therefore, differences between males and females were not statistically tested. For all raw data, see S1 File.

## Results

We investigated diurnal variation of a set of markers (relevant for metabolic disorders and hormonal cancers) in a routine setting in males and females. To determine the diurnal variation we performed two analyses: 1) Cosine fitting analysis: using CircWave software we determined whether the level of the marker follows a cosine rhythm; 2) RM-ANOVA analysis: determine effects of time of day on parameter levels. If analysis 1, analysis 2 or both indicated that significant variation was present we identified the markers as having diurnal variation in our experiment.

### Hormones

**Fig 1:**
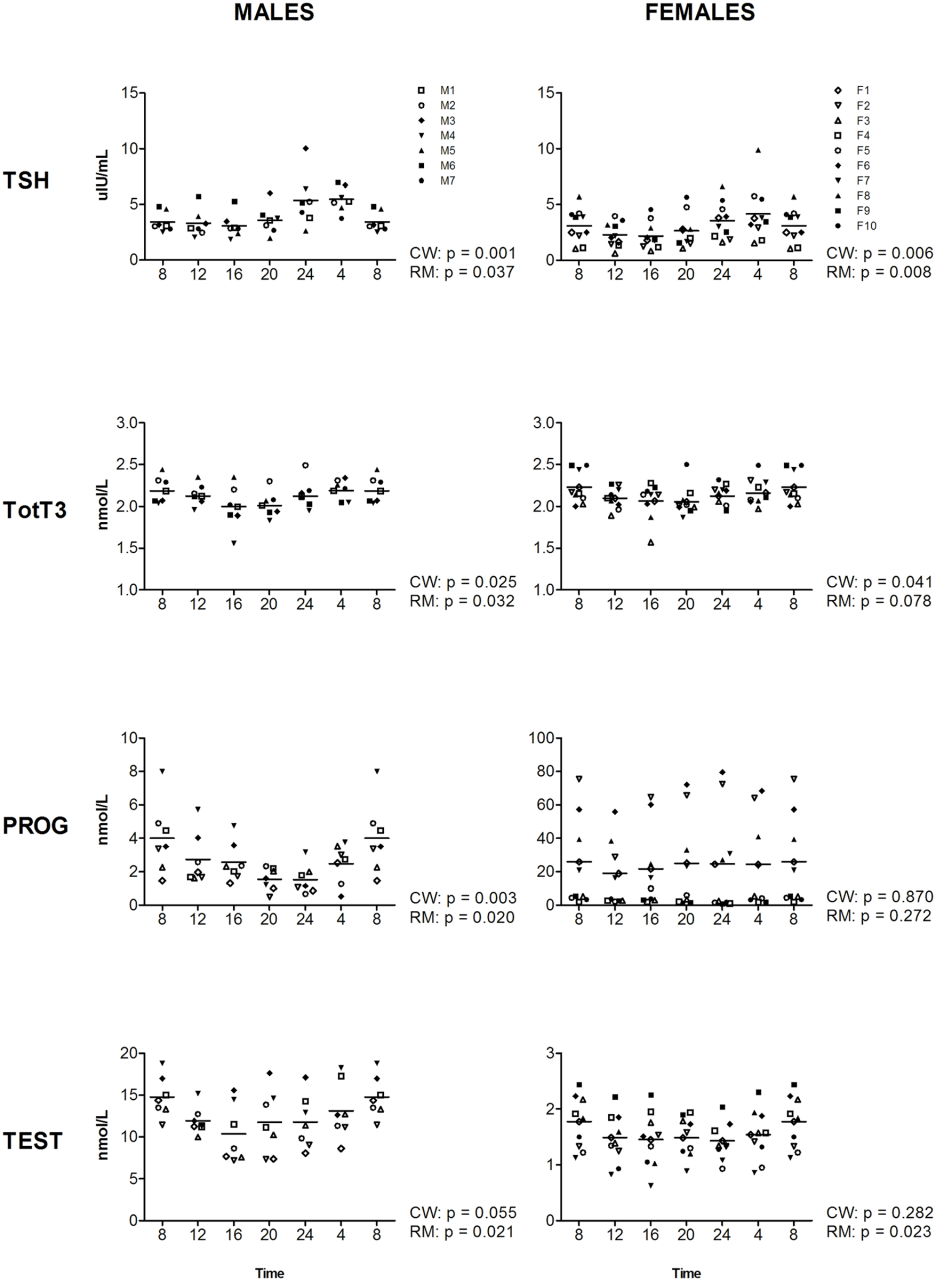
Levels of TSH, TotT3, PROG, and TEST during the day, measured at four-hour intervals for male (left panels) and female (right panels) volunteers. Time indicates clock time. Values at 8-hr were double plotted to help visualize daily patterns. Data represent mean ±sd.

**Fig 2:**
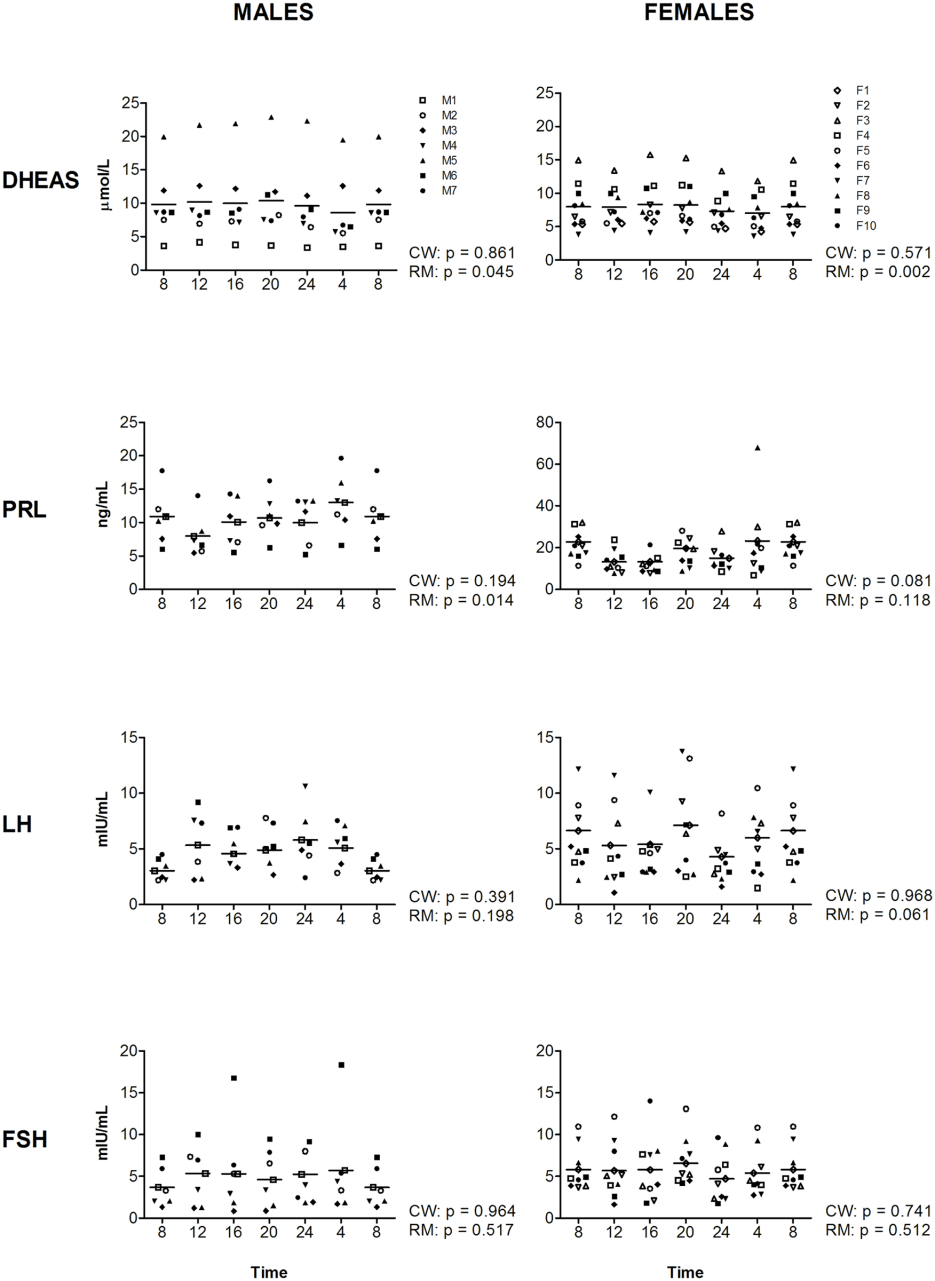
Levels of DHES, PRL, LH, and FSH during the day, measured at four-hour intervals for male (left panels) and female (right panels) volunteers. Time indicates clock time. Values at 8-hr were double plotted to help visualize daily patterns. Data represent mean ±sd.

**Fig 3:**
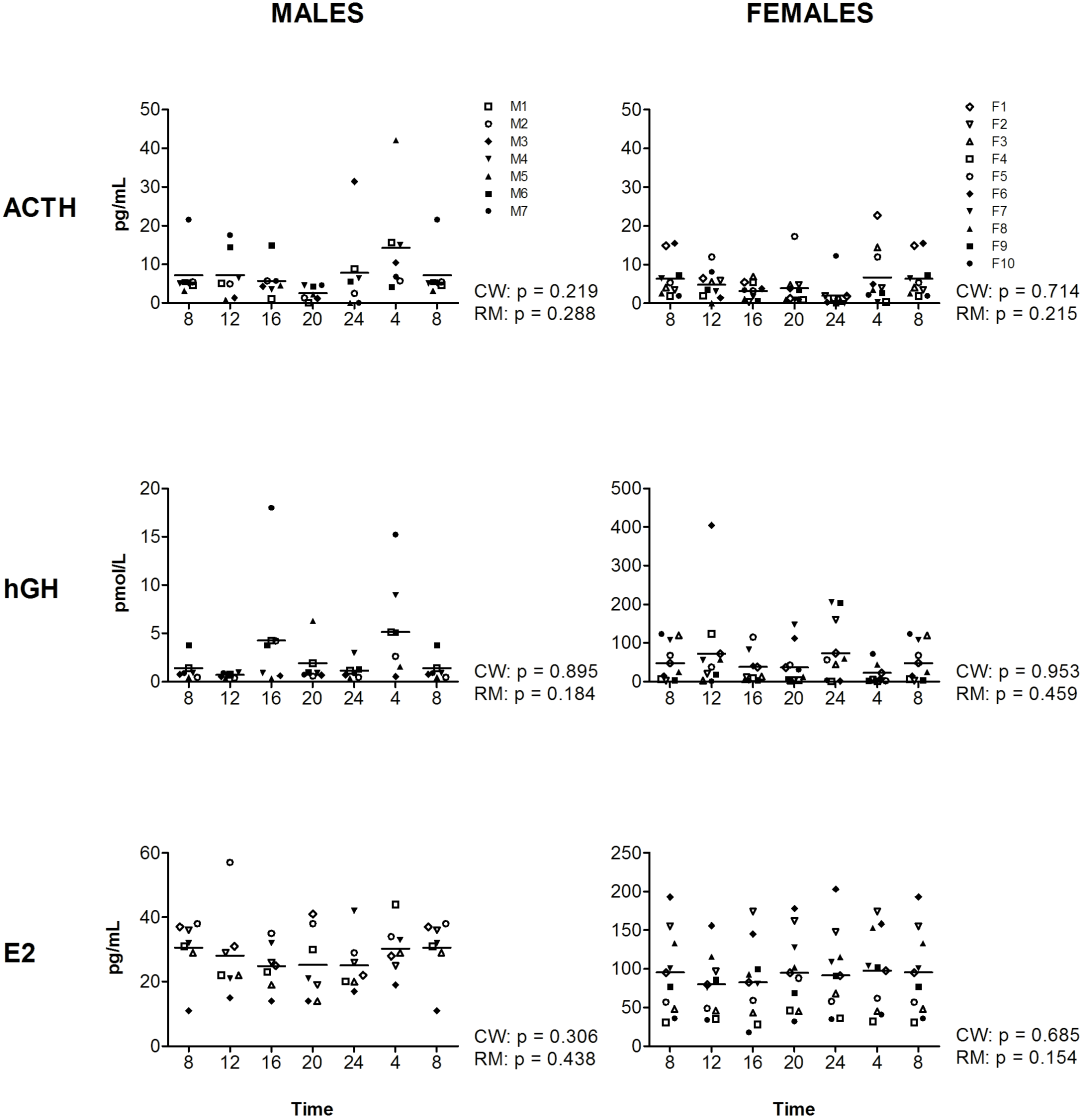
Levels of ACTH, hGH, and E2 during the day, measured at four-hour intervals for male (left panels) and female (right panels) volunteers. Time indicates clock time. Values at 8-hr were double plotted to help visualize daily patterns. Data represent mean ± sd.

Cosine analysis showed that for two of the eleven investigated hormonal markers, a significant cosine curve fit was present in both genders: thyroid-stimulating hormone (TSH) and total triiodothyronine (totT3) (Fig 1). The levels of TSH are highest during the middle of the night, where in males (m) the peak is observed slightly earlier (Clock Time (CT) = 01:54, p = 0.001, amplitude as percentage of the median = 75.89%) compared to females (f) (CT = 03:32, p = 0.006, amplitude = 74.26%). Levels of totT3 are highest at the beginning of the day (m: CT = 06:18, p 0.025, amplitude = 10.14%; f: CT = 07:04, p = 0.041, amplitude = 8.08%). Progesterone (PRG) had a significant cosine curve fit in males, but not in females (m: CT = 07:52, p = 0.003, amplitude = 117.78%; f: p = 0.870) (Fig 1). For testosterone (TEST), a trend towards a cosine curve fit was present in males, but not females (m: CT = 07:33, p = 0.055, amplitude = 38.38%; f: p = 0.282) (Fig 1). For a complete overview of CircWave analyses for all 11 hormonal markers, see Figs 1–3 and S3 Table.

For the two markers with a significant cosine fit in both genders, TSH and TotT3, an effect of time was observed with RM-ANOVA as well (for statistics see S3 Table), although for TotT3 in females only a trend is observed (p = 0.078). In addition, for four markers where a cosine curve could not significantly be fitted, a significant effect of time was observed, for TEST, DHEAS, PRG and prolactin (PRL) (Figs 1 and 2). RM-ANOVA shows that TEST and DHEAS are present at different levels during the 6 time-points in both genders (TEST: m: p 0.021, f: p = 0.023; DHEAS: m: p = 0.045, f: p = 0.002). Furthermore, PRL and PRG are significantly different over the time-points in one gender: PRL (m: p = 0.014, f: p = 0.118) and PRG (m: p = 0.020, f: p = 0.272). For complete overview of the results of the RM-ANOVA on all time-points, see Figs 1–3 and S3 Table.

### Lipids

**Fig 4:**
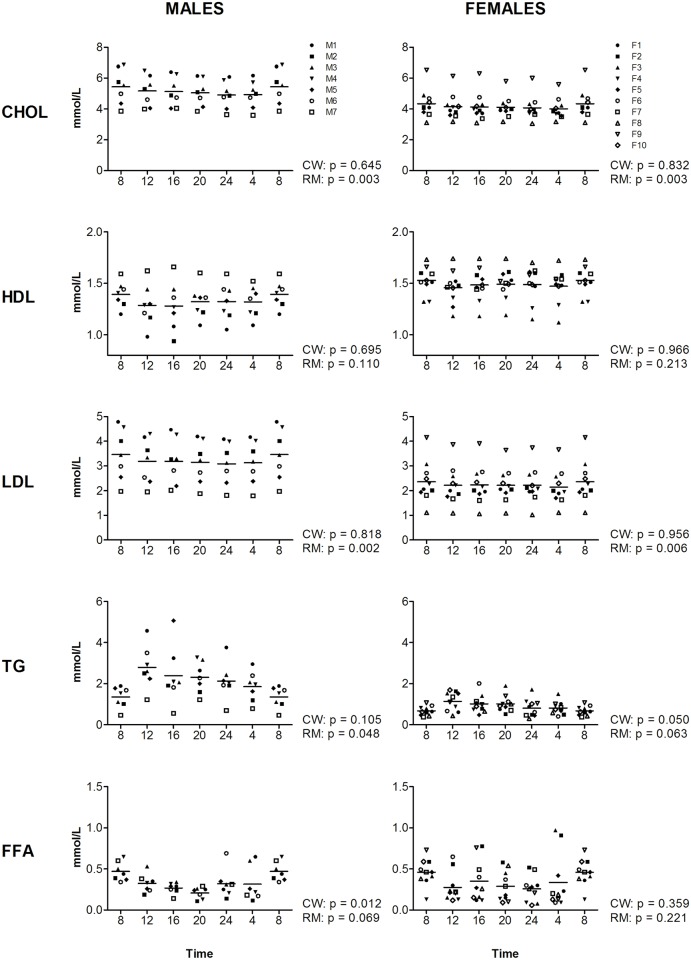
Levels of different lipids during the day, measured at four-hour intervals for male (left panels) and female (right panels) volunteers. Time indicates clock time. Values at 8-hr were double plotted to help visualize daily patterns. Data represent mean ±sd.

Five types of lipids were examined; free fatty acids (FFA), triglycerides (TG), total cholesterol (CHOL), low-density lipoprotein (LDL), and high-density lipoprotein (HDL) (Fig 4). CircWave analysis indicated that FFA levels follow a cosine curve in males (p = 0.012, amplitude 74.91%), but not significantly in females (p = 0.359, amplitude = 80.70%). The levels of FFA were highest at the beginning of the active period in males (CT = 08:31). A trend towards a cosine curve fit was observed for TG in females (p = 0.050, amplitude = 50.33%), but not in males (p = 0.105, amplitude = 70.36%) (Fig 4). Visual inspection of the levels of FFA and TG in Fig 4 implies that the differences between males and females for these markers is likely related to the variation observed within the groups, since the highest levels are observed at similar time-points for both genders. For complete overview of all markers, see Fig 4 and S4 Table.

Despite the limited presence of significant cosine curve fits observed among the different lipid markers, for several markers RM-ANOVA shows significant effects of time: for CHOL and LDL in both genders (CHOL: m: p = 0.003, f: p = 0.003; LDL: m: p = 0.002, f: p = 0.006), for TG in males and a trend was present in females (m: p = 0.048, f: p = 0.063) (S4 Table). The levels of FFA show a trend towards an effect of time in males (m: p = 0.069, f: p = 0.221). For HDL, no significant effect was observed in any gender (m: p = 0.110, f: p = 0.213). The highest level of most lipids including FFA, LDL, and CHOL was observed at the beginning of the active phase, the highest level of TG in males was observed during the middle of the day (Fig 4).

In summary, for all lipid markers, except HDL significant or trends towards diurnal variation are observed.

### Classical circadian markers

**Fig 5:**
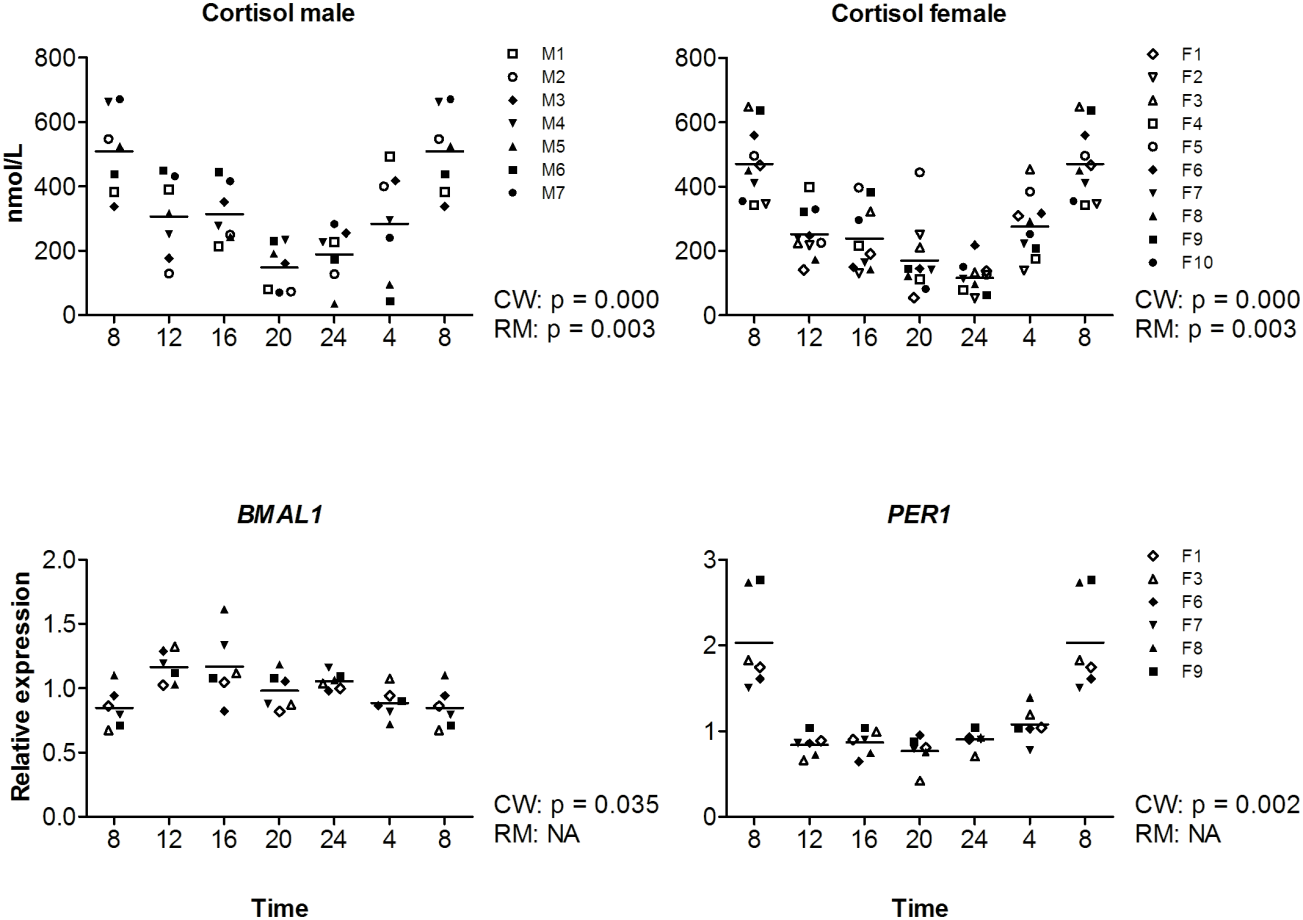
Around the clock levels for classical circadian markers cortisol, *PER1* and *BMAL1*. Upper panels show cortisol levels in male (left panels) and female (right panels) volunteers, determined in serum. mRNA expression of *BMAL1* and *PER1* in buffy coats are depicted in the lower panels (females subset n = 6). All were measured at 4 hour intervals, where time indicates clock time. Values at 8-hr were double plotted to help visualize daily patterns. Data represent mean ±sd.

The levels of cortisol were investigated in all participants (n = 17), clock genes *PER1* and *BMAL1* were investigated in a subset (n = 6, females) to gain insight in classical circadian markers in uncontrolled conditions (Fig 5). Cortisol levels show a significant cosine curve fit, are highest at the beginning of the day (m: CT = 07:47; f: CT = 07:29), and the rhythm has a relative large amplitude (m: p < 0.0005, amplitude = 129.11%; f: p < 0.0005, amplitude = 161.63%) (Fig 5, upper panels). The levels of *PER1* and *BMAL1* mRNA show a significant cosine curve fit as well (*PER1* p = 0.002, amplitude = 80.15%; *BMAL1* p = 0.035, amplitude = 35.55%). Levels of *PER1* were highest during the morning (CT = 8:07), while levels of *BMAL1* were highest during the afternoon (CT = 14:12). For complete overview of statistics see S5 Table.

## Discussion

The aim of the present study was to investigate diurnal variation in a set of hormones, metabolic markers, and classical circadian markers in serum, plasma and peripheral blood mononuclear cells (PBMCs), in a routine non-fasted molecular epidemiology-like setting, with no restrictions on sleep or diet.

### Hormones

#### Markers without diurnal variation

For markers without diurnal variation, taking the time-point of sampling into account is not required. This study observed no diurnal variation or trends towards diurnal variation in any gender in four of the eleven hormonal markers investigated, ACTH, hGH, E2, and FSH. Previous published studies performed in a different type of setting than the present study, namely a controlled (laboratory) settings do show diurnal variation in ACTH levels in males, with the highest level being observed at CT 4.00 h [16] or CT 7.00 h [22], whereas no diurnal was observed in females [22]. Interestingly, in the present study, levels were also highest at CT 4.00, indicating similar patterns. However, in our study larger amount of variation is expected than a controlled laboratory setting potentially causing the diurnal variation of ACTH undetectable within the inter-individual variations. Since during day time-points, when sampling is usually performed in a cohort study, variation in ACTH levels is minimal. Thus, diurnal variation seems not to be a major issue for ACTH in a molecular epidemiology setting. However, considering the previous studies on ACTH and that in a large cohort setting power will be increased, diurnal variation might become larger than the inter-individual variation and caution is required. In addition, if subjects are investigated which possibly have a circadian disruption (e.g. night shift workers), variation in levels of the markers during night time-points should be taken into account as well (see recommendations section for suggestions).

For hGH, former reports in controlled settings have shown diurnal variation in males [22, 23]. However, it is important to note that it is known that hGH is released in pulses with levels varying up to 4 times [24]. Hence, in the present study with sampling intervals of 4 hours these pulse releases might interfere with the detection of diurnal variation. Indeed, in the present study at several time-points large variations in the levels of hGH are observed, particularly in males. This indicates that hGH is a difficult marker to measure in a non-time-controlled cohort setting, since possible differences are easily masked by the large amount of variation present for this marker.

For estradiol, the absence of diurnal variation in the present study is in line with previous studies that reported the absence of diurnal variation in females [25, 26], although diurnal variation is observed in estradiol levels in elderly females (mean age 77 years) [22]. Together, these data indicate that in a cohort study with non-elderly adults, diurnal variation of estradiol is not an issue.

For FSH, conflicting data have been published previously. In females, the absence of a diurnal variation was reported in studies using (mildly) controlled settings [15, 22], while a third study reported diurnal variation in females during different phases of the menstrual cycle, with the highest levels of FSH being observed during the afternoon [26]. For males, conflicting results have been reported as well: a study by Spratt et al. showing the absence of diurnal variation in males [27], while Nicolau et al., reported the presence of a diurnal variation in elderly males [22]. Possibly, age has a role in the diurnal variation of FSH. However, the present study indicates that in a routine setting, time of day is not a major source of variation for FSH levels.

#### Markers with diurnal variation

For eight of the twelve investigated hormonal markers, diurnal variation was observed in at least one gender. Markers with the most robust diurnal variation in both genders were TSH and T3. This is in line with previous findings for these markers [15, 22, 23, 28]. For DHEAS, a previous study reported circadian rhythmicity in males and females [22], however, in the present study the rhythm of DHEAS could not be fitted to a cosine curve. The levels of DHEAS differed among the time-points and the pattern of expression (e.g. highest levels in the afternoon) is comparable to the previously published results, confirming diurnal variation of DHEAS.

For testosterone, diurnal variation was not robust, but significant changes were observed when the time-points were compared in both genders. Diurnal variation in testosterone is in line with previous findings [22, 25, 27, 29–31]. The study by Spratt et al. showed that the robustness of the diurnal variation of testosterone was largely dependent on the frequency of sampling.

For several of the other markers, the robustness of diurnal variation varied among genders. Diurnal variation was often more pronounced in males. Since in the present study menstrual period was not assessed, nor was the use of contraceptives, this might explain the less robust results in females. For example, progesterone showed diurnal variation in males, but not females. For females, it has been shown that the rhythmicity of progesterone varies with the menstrual period, including the timing of the highest levels [22, 32, 33]. Interestingly, 5 females show high progesterone levels, which are indicative of the lutheal phase. Of these females, two show a significant or trend (p-value between 0.05 and 0.1) towards an individual cosine curve fit (#6: p = 0.0059 and #7: p = 0.05222). These results suggest that indeed variation in menstrual period, and possibly contraceptives use, interfere with measurements of progesterone in a cohort setting.

For the final two markers, LH and PRL, only mild amounts of diurnal variation were observed. For LH previous studies in females have shown that rhythmicity is dependent on the menstrual cycle and that peak timing differs among women [15, 26, 33], which likely explains the results of the present study. For prolactin, in the present study only a trend towards a cosine curve fit was observed in females and effects of time (by RM-ANOVA) in males. This is in line with previous studies that have shown diurnal variation of prolactin in females in controlled settings [32, 34], and higher levels of prolactin during the night in males [35]. Together, these results indicate that for prolactin time of day should be taken into account (see recommendations section for suggestions).

### Lipids

Of the 5 lipid markers, only HDL showed no diurnal variation (no cosine curve fit and no effect of time by RM-ANOVA). Previously, it has been shown that a large subset of lipids shows diurnal variation for examples see references: [36, 37], for review see reference: [38], however, the rhythmicity of these markers is highly variable among individuals [37]. Furthermore, it is known that many lipids are directly regulated by the endogenous circadian clock [20, 21, 38], lipid metabolism is related to sleep [19, 39], and that levels of FFA, TG and LDL are dependent on meal-timing [40, 41] The results of the present study, diurnal variation in LDL, TG, FFA and total cholesterol are thus in line with previous findings. These results show that for these markers time of day needs to be taken into account (see recommendations section below for suggestions). Previously, diurnal variation has been observed for HDL in a controlled setting [36], however, our study indicates that this variation does not exceed the inter-individual variation in a routine setting.

### Classical circadian markers

In chronobiology research, several markers are often used to study circadian rhythm. Hence, these can be named as ‘classical circadian markers’. One of these markers is cortisol, which is often used to study circadian rhythm in humans. In addition, components of the molecular biological clock can be used to study circadian rhythm, i.e. ‘clock genes’. Since these genes are part of the core clock mechanism in cells, their rhythm reflects the circadian rhythm in these cells. We show that the ‘classical circadian markers’ investigated in this study (cortisol and the clock genes *PER1* and *BMAL1*) have a robust cosine curve fit in our study population, which resembles a routine non-fasted molecular epidemiology setting. Our findings for cortisol (rhythmic expression with high levels during the early morning), are in line with previous literature. Multiple studies, in a range of different settings, have reported similar findings (for review see reference [12], for examples see references [42–44]).

In our study *PER1* expression fits to a cosine curve with the highest levels in the morning, which is also consistent with previous studies. For example, James et al. observed rhythmic *PER1* expression with peak levels on average 2:36 h after waking (±1:47 h) in PBMCs [18], Kusanagi et al. observed rhythmic *PER1expression* with peak expression on average at 7:42 h in PMBCs [42], and Takimoto et al. reported peak levels at 06:00 h in whole blood cells [43]. For *BMAL1*, previous studies have indicated that rhythmicity of *BMAL1* gene expression in PBMCs is highly variable amongst individuals [18, 44]. For example, *BMAL1* expression has been reported to be high during the middle of the day (comparable to our study) [18, 44], during the night [43], during the evening [44], or not being rhythmic [42]. Teboul et al. suggested that this variation might be related to chronotype, since in their study two groups of individuals could be distinguished: peak expression of BMAL1 during the middle of the day (12:30 hr) or during the evening (21:45 hr).

Together, our results shows that thediurnal variation, including the acrophase of the fitted cosine curves, of several classical markers in our study is comparable to previous studies using (mildly) controlled settings [18, 42–44].

### Concluding remarks and reccomendations

The present study showed that markers for which diurnal variation (circadian rhythms and/or time-of-day-effects) is not an issue in a routine setting are ACTH, FSH, E2 and HDL. For all other tested markers diurnal variation was observed, which exceeded the inter-individual variation in a routine setting. It is important to note that we tested this in a small test group (n = 17). In a large cohort with numerous subjects, levels of inter-individual variation might decrease due to larger sample sizes, making the contribution of diurnal variation relatively larger. Hence, for some of the markers without detectable diurnal variation in the present study, diurnal variation might play a role in larger studies. Nevertheless, our study provides an indication for which markers diurnal variation needs to be taken into account in a routine setting.

Taking diurnal variation into account can be done in several ways depending on the study design and marker characteristics (such as dependent on food intake, sleep/wake cycle etc.). For example, measurements can be taken at a single time-point or if sampling at a single time-points is not possible, registration of sampling time and their relevant time-points (e.g. timing of food intake) is an alternative method to take into account diurnal variation. Apart from a research setting, several of the markers in our study are measured in a clinical setting as well. In general, a substantial amount of variance is incorporated in reference levels for diagnostic purposes. However, for biomarkers with large diurnal variation it might be beneficial to take multiple samples during the day or define reference levels that take into account time-of-day.

Our finding that most markers tested show diurnal variation is in line with recent studies showing that many genes are regulated by the circadian clock. For example, a recent study has shown that 43% of the protein coding genes shows diurnal variation in at least one organ in mice [45], as a consequence one could expect diurnal variation in many serum or plasma protein levels as well. This large set of markers with diurnal variation indicates the need for investigating diurnal variation before setting up studies measuring markers in routine and controlled settings, since time- of- day likely matters for many more markers than the ones investigated in the present study.

## Supporting Information

S1 File Raw data files of data presented in this study. (XLSX)

S1 Table Participant characteristics. Age is provided in years (y), weight in kilograms (kg), waist circumference in centimeters (cm), total sleep duration between the first sampling time at 08:00 AM and the last sampling time at 04:00 AM in hours (hr), sleep timing of sleep episodes between the first sampling time at 08:00 AM and the last sampling time at 04:00 AM provided in clock time (CT).
(DOCX)

S2 Table Overview of methods used to determine parameters in plasma and serum. The intra assay variation (CV) was determined with three quality control samples (N = 8) for the markers as determined on the auto-analyzer and with two quality control samples (N = 5) for markers as measured on the immune-analyzer. The CVs of markers measured with the Luminex technique were obtained from the manufacturer.
(DOCX)

S3 Table Overview of hormonal parameters in serum or plasma for A. CircWave analysis of cosine curve fits; B. Repeated-Measures ANOVA to determine effects of time. Amplitude is presented as the % of the median. Ct = Clock time.
(DOCX)

S4 Table Overview of lipid parameters in serum or plasma for A. CircWave analysis of cosine curve fits; B. Repeated-Measures ANOVA to determine effects of time. Amplitude is presented as the % of the median. Ct = Clock time.
(DOCX)

S5 Table CircWave analysis of classical circadian markers in serum (CORT) or PBMCs (*PER1*, *BMAL1*). Amplitude is presented as the % of the median. Ct = Clock time.
(DOCX)
