# Supplementary material for: Diurnal Variation of Hormonal and Lipid Biomarkers in a Molecular Epidemiology-Like Setting
Source: PLoS One. 2015 Aug 18;10(8):e0135652. doi: 10.1371/journal.pone.0135652 (PMC4540433; doi:10.1371/journal.pone.0135652)
Supplement: S1 Table — Age is provided in years (y), weight in kilograms (kg), waist circumference in centimeters (cm), total sleep duration between the first sampling time at 08:00 AM and the last sampling time at 04:00 AM in hours (hr), sleep timing of sleep episodes between the first sampling time at 08:00 AM and the last sampling time at 04:00 AM provided in clock time (CT). (DOCX) [file pone.0135652.s002.docx]

**Supplementary data Table S1.** Participant characteristics. Age is provided in years (y), weight in kilograms (kg), waist circumference in centimeters (cm), total sleep duration between the first sampling time at 08:00 AM and the last sampling time at 04:00 AM in hours (hr), sleep timing of sleep episodes between the first sampling time at 08:00 AM and the last sampling time at 04:00 AM provided in clock time (CT).

| Participant | Gender | Age (y) | Weight (kg) | Waist circumference (cm) | Total sleep duration (hr) between 08:00 and 04:00 | Sleep timing (CT) between 08:00 and 04:00 |
| --- | --- | --- | --- | --- | --- | --- |
| F1 | f | 22 | 85 | 80 | 1.25 | 02:00-03:15 |
| F2 | f | 22 | 52 | 68 | 5.42 | 13:30-14:30, 22:00-23:30, 00:45-3:40 |
| F3 | f | 22 | 50 | 83 | 3.00 | 00:15-03:15 |
| F4 | f | 22 | 49 | 69 | 3.00 | 00:15-03:15 |
| F5 | f | 22 | 48 | 67 | 3.00 | 00:15-03:15 |
| F6 | f | 22 | 50 | 69 | 3.00 | 00:15-03:15 |
| F7 | f | 21 | 59 | 75 | 6.50 | 09:00-10:00, 17:00-19:00, 00:15-03:45 |
| F8 | f | 21 | 55 | 77 | 4.75 | 16:15-18:00, 00:15-03:15 |
| F9 | f | 21 | 82 | 93 | 0.00 | none |
| F10 | f | 22 | 55 | 74 | 2.50 | 00:30-03:00 |
| M1 | m | 41 | 105 | 123 | 4.00 | 14:00:15:30, 00:30-03:00 |
| M2 | m | 24 | 100 | 110 | 3.00 | 00:30-03:30 |
| M3 | m | 23 | 72 | 86 | 2.00 | 01:00-03:00 |
| M4 | m | 21 | 70 | 83 | 5.00 | 20:30-23:30, 01:00-03:00 |
| M5 | m | 21 | 88 | 85 | 3.00 | 00:15-03:15 |
| M6 | m | 23 | 79 | 79 | 3.33 | 00:30-03:50 |
| M7 | m | 21 | 75 | 90 | 3.67 | 00:10-03:50 |
| Females |  |  |  |  |  |  |
| Mean |  | **21.70** | **58.50** | **75.50** | **3.24** |  |
| SD |  | **0.48** | **13.61** | **8.17** | **1.91** |  |
|  |  |  |  |  |  |  |
| Males |  |  |  |  |  |  |
| Mean |  | **24.86** | **84.14** | **93.71** | **3.43** |  |
| SD |  | **7.22** | **13.90** | **16.35** | **0.94** |  |
