# Supplementary material for: Diurnal Variation of Hormonal and Lipid Biomarkers in a Molecular Epidemiology-Like Setting
Source: PLoS One. 2015 Aug 18;10(8):e0135652. doi: 10.1371/journal.pone.0135652 (PMC4540433; doi:10.1371/journal.pone.0135652)
Supplement: S2 Table — The intra assay variation (CV) was determined with three quality control samples (N = 8) for the markers as determined on the auto-analyzer and with two quality control samples (N = 5) for markers as measured on the immune-analyzer. The CVs of markers measured with the Luminex technique were obtained from the manufacturer. (DOCX) [file pone.0135652.s003.docx]

**Supplementary Table S2.** Overview of methods used to determine parameters in plasma and serum. The intra assay variation (CV) was determined with three quality control samples (N=8) for themarkers as determined on the auto-analyzer and with two quality control samples (N=5) for markers as measured on the immune-analyzer. The CVs of markers measured with the Luminex technique were obtained from the manufacturer.

|  | Technique | CV | Source |
| --- | --- | --- | --- |
| ACTH | Luminex (Millipore) | 10.8 | Plasma |
| CORT | Immune--analyzer (Access-2, Beckman-Coulter) | 6.2 | Serum |
| DHEAS | Immune--analyzer (Access-2, Beckman-Coulter) | 3.9 | Serum |
| E2 | Immune--analyzer (Access-2, Beckman-Coulter) | 6.3 | Serum |
| FSH | Luminex (Millipore) | 7.2 | Plasma |
| hGH | Immune--analyzer (Access-2, Beckman-Coulter) | 2.3 | Serum |
| LH | Luminex (Millipore) | 6.3 | Plasma |
| PRL | Immune--analyzer (Access-2, Beckman-Coulter) | 1.8 | Serum |
| PRG | Immune--analyzer (Access-2, Beckman-Coulter) | 10.8 | Serum |
| TEST | Immune--analyzer (Access-2, Beckman-Coulter) | 1.4 | Serum |
| TotT3 | Immune--analyzer (Access-2, Beckman-Coulter) | 4.2 | Serum |
| TSH | Immune--analyzer (Access-2, Beckman-Coulter) | 1.4 | Serum |
| FFA | Auto-analyzer (Unicel DxC 800, Beckman-Coulter) | 4.4 | Plasma |
| HDL | Auto-analyzer (Unicel DxC 800, Beckman-Coulter) | 4.0 | Plasma |
| LDL | Auto-analyzer (Unicel DxC 800, Beckman-Coulter) | 7.2 | Plasma |
| TG | Auto-analyzer (Unicel DxC 800, Beckman-Coulter) | 4.3 | Plasma |
| CHOL | Auto-analyzer (Unicel DxC 800, Beckman-Coulter) | 4.5 | Plasma |
