# Supplementary material for: Diurnal Variation of Hormonal and Lipid Biomarkers in a Molecular Epidemiology-Like Setting
Source: PLoS One. 2015 Aug 18;10(8):e0135652. doi: 10.1371/journal.pone.0135652 (PMC4540433; doi:10.1371/journal.pone.0135652)
Supplement: S3 Table — Amplitude is presented as the % of the median. Ct = Clock time. (DOCX) [file pone.0135652.s004.docx]

**Supplementary Table S3.** Overview of hormonal parameters in serum or plasma for **A.** CircWave analysis of circadian rhythms; **B.** Repeated-Measures ANOVA to determine daily variation. Amplitude is presented as the % of the median. Ct = Clock time.

| 1. **Circwave analysis** | | | | | | | |
| --- | --- | --- | --- | --- | --- | --- | --- |
|  | **males** | | | **females** | | | |
| **Markers** | **p-value** | **peak (CT)** | **amplitude** | | **p-value** | **peak (CT)** | **amplitude** |
| ACTH | 0.219 | 17:54 | 6.12% | | 0.714 | 17:22 | 3.71% |
| CORT | 0.000 | 07:47 | 129.11% | | 0.000 | 07:29 | 161.63% |
| DHEAS | 0.861 | 20:13 | 21.89% | | 0.571 | 18:23 | 21.36% |
| E2 | 0.306 | 06:51 | 24.66% | | 0.685 | 05:52 | 25.11% |
| FSH | 0.964 | 02:03 | 40.47% | | 0.741 | 18:41 | 31.20% |
| hGH | 0.895 | 04:13 | 581.23% | | 0.953 | 11:31 | 397.11% |
| LH | 0.391 | 00:36 | 59.22% | | 0.968 | 07:15 | 45.25% |
| PRL | 0.194 | 04:55 | 49.11% | | 0.081 | 06:20 | 97.09% |
| PRG | 0.003 | 07:52 | 117.78% | | 0.870 | 06:17 | 44.70% |
| TEST | 0.055 | 07:33 | 38.38% | | 0.282 | 07:38 | 22.75% |
| TotT3 | 0.025 | 06:18 | 10.14% | | 0.041 | 07:04 | 8.08% |
| TSH | 0.001 | 01:54 | 75.89% | | 0.006 | 03:22 | 74.26% |

| 1. **RM-ANOVA all time points** | | | | |
| --- | --- | --- | --- | --- |
|  | **males** | | **females** | |
| **Markers** | **p-value** | **F-value** | **p-value** | **F-value** |
| ACTH | 0.288 | F (2.277, 13.66) = 1.375 | 0.215 | F (3.037, 27.33) = 1.775 |
| CORT | 0.003 | F (2.611, 15.67) = 7.614 | 0.000 | F (3.466, 31.19) = 20.56 |
| DHEAS | 0.045 | F (2.389, 14.33) = 3.694 | 0.002 | F (2.943, 26.49) = 6.530 |
| E2 | 0.438 | F (2.760, 16.56) = 0.938 | 0.154 | F (3.873, 34.85) = 1.792 |
| FSH | 0.517 | F (1.990, 11.94) = 0.695 | 0.512 | F (2.079, 18.71) = 0.687 |
| hGH | 0.184 | F (1.465, 8.792) = 2.091 | 0.459 | F (2.243, 20.18) = 0.838 |
| LH | 0.198 | F (2.794, 16.76) = 1.746 | 0.061 | F (3.491, 31.42) = 2.617 |
| PRL | 0.014 | F (2.715, 16.29) = 4.984 | 0.118 | F (1.865, 16.79) = 2.461 |
| PRG | 0.020 | F (2.310, 13.86) = 4.974 | 0.272 | F (2.204, 19.84) = 1.395 |
| TEST | 0.021 | F (2.945, 17.67) = 4.221 | 0.023 | F (2.760, 24.84) = 3.922 |
| TotT3 | 0.032 | F (2.461, 14.77) = 4.078 | 0.078 | F (2.896, 26.06) = 2.570 |
| TSH | 0.037 | F (1.266, 7.596) = 5.991 | 0.008 | F (1.700, 15.30) = 7.305 |
