# Supplementary material for: Diurnal Variation of Hormonal and Lipid Biomarkers in a Molecular Epidemiology-Like Setting
Source: PLoS One. 2015 Aug 18;10(8):e0135652. doi: 10.1371/journal.pone.0135652 (PMC4540433; doi:10.1371/journal.pone.0135652)
Supplement: S4 Table — Amplitude is presented as the % of the median. Ct = Clock time. (DOCX) [file pone.0135652.s005.docx]

**Supplementary Table S4.** Overview of lipid parameters in serum or plasma for **A.** CircWave analysis of circadian rhythms; **B.** Repeated-Measures ANOVA to determine daily variation. Amplitude is presented as the % of the median. Ct = Clock time.

| 1. **Circwave analysis** | | | | | | | |
| --- | --- | --- | --- | --- | --- | --- | --- |
|  | **males** | | | **females** | | | |
| **Markers** | **p-value** | **peak (CT)** | **amplitude** | | **p-value** | **peak (CT)** | **amplitude** |
| FFA | 0.012 | 08:31 | 74.91% | | 0.36 | 06:56 | 80.70% |
| HDL | 0.695 | 07:38 | 9.20% | | 0.97 | 07:30 | 3.86% |
| LDL | 0.818 | 08:07 | 12.02% | | 0.96 | 08:46 | 8.35% |
| TG | 0.105 | 13:46 | 70.36% | | 0.05 | 14:13 | 50.33% |
| CHOL | 0.645 | 08:47 | 11.44% | | 0.83 | 08:57 | 8.17% |

| 1. **RM-ANOVA all time points** | | | | | |
| --- | --- | --- | --- | --- | --- |
|  | **males** | | **females** | | |
| **Markers** | **p-value** | **F-value** | | **p-value** | **F-value** |
| FFA | 0.069 | F (2.442, 14.65) = 3.078 | | 0.221 | F (2.638, 23.75) = 1.589 |
| HDL | 0.110 | F (2.708, 16.25) = 2.399 | | 0.213 | F (2.475, 22.28) = 1.641 |
| LDL | 0.002 | F (2.875, 17.25) = 7.746 | | 0.006 | F (2.660, 23.94) = 5.670 |
| TG | 0.048 | F (1.943, 11.66) = 4.012 | | 0.063 | F (2.846, 25.62) = 2.798 |
| CHOL | 0.003 | F (2.381, 14.29) = 8.618 | | 0.003 | F (2.938, 26.44) = 6.020 |
