# Supplementary material for: Diurnal Variation of Hormonal and Lipid Biomarkers in a Molecular Epidemiology-Like Setting
Source: PLoS One. 2015 Aug 18;10(8):e0135652. doi: 10.1371/journal.pone.0135652 (PMC4540433; doi:10.1371/journal.pone.0135652)
Supplement: S5 Table — Amplitude is presented as the % of the median. Ct = Clock time. (DOCX) [file pone.0135652.s006.docx]

**Supplementary Table S5.** CircWave analysis of circadian rhythms classical circadian markers in serum (CORT) or PBMCs (*PER1, BMAL1*). Amplitude is presented as the % of the median. Ct = Clock time.

| **Circwave analysis** | | | | | | | |
| --- | --- | --- | --- | --- | --- | --- | --- |
|  | **males** | | | **females** | | | |
| **Markers** | **p-value** | **peak (CT)** | **amplitude** | | **p-value** | **peak (CT)** | **amplitude** |
| CORT | 0.000 | 07:47 | 129.11% | | 0.000 | 07:29 | 161.63% |
| *PER1* |  |  |  | | 0.002 | 08:07 | 80.15 % |
| *BMAL1* |  |  |  | | 0.035 | 14:12 | 35.55 % |
